# Assessment of visual disability using visual evoked potentials

**DOI:** 10.1186/1471-2415-12-36

**Published:** 2012-08-06

**Authors:** Jihoon Jeon, Seiyul Oh, Sungeun Kyung

**Affiliations:** 1 Department of ophthalmology, Dankook University Hospital, 359 Manghang-Ro, Dongnam-Gu, Cheonan-City, Chungchungnam-Do, South Korea; 2 Department of Ophthalmology, Sungkyunkwan University School of Medicine, Samsung Medical Center, 50 Ilwon-dong, Gangnam-gu, Seoul city, South Korea

**Keywords:** Pattern visual evoked potentials (VEP), Disability assessment, Objective visual acuity, Malingering

## Abstract

**Background:**

The purpose of this study is to validate the use of visual evoked potential (VEP) to objectively quantify visual acuity in normal and amblyopic patients, and determine if it is possible to predict visual acuity in disability assessment to register visual pathway lesions.

**Methods:**

A retrospective chart review was conducted of patients diagnosed with normal vision, unilateral amblyopia, optic neuritis, and visual disability who visited the university medical center for registration from March 2007 to October 2009. The study included 20 normal subjects (20 right eyes: 10 females, 10 males, ages 9–42 years), 18 unilateral amblyopic patients (18 amblyopic eyes, ages 19–36 years), 19 optic neuritis patients (19 eyes: ages 9–71 years), and 10 patients with visual disability having visual pathway lesions. Amplitude and latencies were analyzed and correlations with visual acuity (logMAR) were derived from 20 normal and 18 amblyopic subjects. Correlation of VEP amplitude and visual acuity (logMAR) of 19 optic neuritis patients confirmed relationships between visual acuity and amplitude. We calculated the objective visual acuity (logMAR) of 16 eyes from 10 patients to diagnose the presence or absence of visual disability using relations derived from 20 normal and 18 amblyopic eyes.

**Results:**

Linear regression analyses between amplitude of pattern visual evoked potentials and visual acuity (logMAR) of 38 eyes from normal (right eyes) and amblyopic (amblyopic eyes) subjects were significant [y = −0.072x + 1.22, x: VEP amplitude, y: visual acuity (logMAR)]. There were no significant differences between visual acuity prediction values, which substituted amplitude values of 19 eyes with optic neuritis into function. We calculated the objective visual acuity of 16 eyes of 10 patients to diagnose the presence or absence of visual disability using relations of y = −0.072x + 1.22 (−0.072). This resulted in a prediction reference of visual acuity associated with malingering vs. real disability in a range >5.77 μV. The results could be useful, especially in cases of no obvious pale disc with trauma.

**Conclusions:**

Visual acuity quantification using absolute value of amplitude in pattern visual evoked potentials was useful in confirming subjective visual acuity for cutoff values >5.77 μV in disability evaluation to discriminate the malingering from real disability.

## Background

Disability diagnosis is a part of the social security system. Determining eligibility for disability diagnosis is difficult because the process of evaluation is dependent upon pension insurance and private disability insurance benefits. Measurement of visual acuity, visual fields, and extra ocular movement are fundamental primary tests for disability determinations. However, there are no sharp inflections in performance abilities corresponding to specific visual acuity.

The cortical visual evoked potential (VEP) is an established method of assessing visual pathway function. Pattern VEP is generally used for the detection of visual function disability (demyelization disease, optic neuritis, ischemic optic neuropathy, compressive optic neuropathy, and amblyopia) and objective visual acuity assessment (visual function for children, malingering, and psychogenic visual disability) [1,2].

Although previous studies have used pattern VEP for the objective assessment of visual acuity, there is little consensus regarding the interpretation of visual acuity (VA) assessments [3-8]. Because there are large differences in VEP among individuals, previous studies involved comparison of the results for two eyes in the same individual rather than using absolute values of amplitude or latency period [9]. Thus, it is difficult to assess visual function with VEP in cases in which both eyes have visual pathway disturbance or functional visual loss combined with visual pathway disability. The clinical usefulness of pattern visual evoked potentials (PVEP) to determine visual acuity is controversial [6,10-12]. We determined a correlation between the PVEP data and visual acuity. The method was applied to patients with optic neuritis to confirm this correlation. Use of PVEP testing to predict objective visual acuity of disability patients was assessed. The present study was performed to obtain reference numbers for visual acuity testing in disability assessment with visual pathway lesions.

## Methods

### Patients

A retrospective chart review of patients diagnosed with normal vision, unilateral anisometropic/strabismic amblyopia, optic neuritis, and visual disability, who visited the university medical center for registration from March 2007 to October 2009, was conducted. We selected 20 normal subjects (20 right eyes: 10 females, 10 males, ages 9–42 years), 18 unilateral amblyopic subjects (18 amblyopic eyes, ages 19–36 years), 19 patients with optic neuritis (19 eyes: ages 9–71 years), and 10 patients with visual disability having visual pathway lesion. A total of 67 patients with VEP recordings were selected. Institutional review board exemption was obtained from the university medical center, and the design of the study followed the tenets of the Declaration of Helsinki. Informed consent was obtained from the parents or patients to allow inclusion in the study.

Corrected VA was measured with the standard Snellen card by conducting cycloplegic refraction at the time of the first examination. Refractive errors were corrected and the best-corrected visual acuities (BCVA) were recorded. Pupillary light reflexes, biomicroscopic and dilated fundoscopic examinations, and VEP recordings were performed on all subjects.

Subjects with Snellen VA of 20/20 and normal ophthalmoscopic exams were defined as having normal vision. Unilateral amblyopia was defined as a visual acuity difference of more than two lines between the two eyes. Anisometropic, strabismic amblyopia, or both, were included. Subjects presumed to be malingering with amblyopia were excluded from the study at the time of diagnosis by repeated fogging and stereopsis tests [13]. Patients with organic eye disease, a history of intraocular surgery, history of cataract, glaucoma, retinal disorders, or laser treatment were excluded from the amblyopia group. Optic neuritis was diagnosed by examining VA and visual field, color vision tests, VEP test, optic nerve appearance, and/or by magnetic resonance imaging (MRI). Visual disability having visual pathway lesion was evaluated at the time of diagnosis by examining visual field, extraocular movement, color vision tests, VEP test, multifocal electroretinogram (mfERG), fluorescein angiography, and retina and optic nerve appearance. Visual disability with retina disease was excluded if a multifocal electroretinogram (mfERG) and fluorescein angiography (FAG) were abnormal. Visual disability with obvious brain lesion was excluded.

### Recording of visual evoked potentials

In all cases, one skilled test conductor measured VEP (RetiSystem; Roland Consult Instrument GmBH, Wiesbaden, Germany) based on the International Society for Clinical Electrophysiology of Vision recommendations [14]. To examine pattern VEP, subjects were seated in a comfortable posture with their visual acuity corrected using trial lenses, and were instructed to maintain fixation at the center of the stimulus located at a distance of 100 cm on a 20 × 30 cm black-and-white video display monitor. The stimulus consisted of a 16 × 16 lattice with a pattern reversal rate of 1.3 times per second and band filter from 0.5 to 100 Hz. The test was conducted by applying the visual stimulus alternately to both eyes. VEP recording was repeated three times in cases where subject cooperation was poor and in disability assessment. Fixation stability of the eyes was monitored closely by an experienced electrophysiology technician. All VEP recordings were performed under the same conditions.

### Statistical analysis

All measurements were obtained from each participant's clinical records. All data were collected, processed, and analyzed using SPSS software (SPSS Inc., Chicago, IL) and GraphPad Software (GraphPad Software, San Diego, CA). The absolute values of both the latency period (P100) and amplitude of the 20 right eyes in normal patients and 18 unilateral amblyopic eyes were analyzed. VEP amplitude (P1–N2), latency (P100), and visual acuity of unilateral amblyopia and normal subjects were used to determine correlations through linear regression analysis.

Pattern VEP-estimated VA in optic neuritis was derived by substituting the amplitude and latency period of optic neuritis into a correlation function. We compared pattern VEP-estimated VA with subjective VA by analysis of variance (ANOVA) to find if the linear regression predicted the VA. In all analyses, *p* < 0.05 were taken to indicate statistical significance. Linear regression analyses with comparing slopes and intercepts of VEP amplitude (P1–N2), and visual acuity between unilateral amblyopia and normal subjects and optic neuritis subjects, were performed.

Receiver operating characteristic (ROC) curve analysis was performed to obtain a cutoff value of the amplitude predicting visual acuity using relation.

## Results

Retrospective clinical data from 20 normal subjects and 18 amblyopic subjects were obtained (Table 1). The average logMAR Snellen acuity of 20 normal subjects (20 normal right eyes) and 18 amblyopic subjects (18 amblyopic eyes) was 0.47 ± 0.57 logMAR. The average pattern amplitude of 20 normal subjects (20 normal right eye) and 18 amblyopic subjects (18 amblyopic eyes) was 10.52 ± 5.96 μV.

**Table 1:** Clinical characteristics and pattern visual evoked potential results in 18 unilateral amblyopic eyes and 20 normal right eyes

**Age**	**BCVA (logMAR)**	**Amplitude (N1-P1)**	**Latency**	**Diagnosis**
19	1	9.46	110	anisometropic amblyopia
19	0.4	15.6	103	anisometropic amblyopia
21	0.5	9.93	99	anisometropic amblyopia
21	0.4	11.4	99	anisometropic amblyopia
19	0.8	10.6	116	anisometropic amblyopia
19	1	4.34	109	anisometropic amblyopia
24	1	3.72	107	anisometropic amblyopia
19	1	0.26	98	anisometropic amblyopia
33	1.4	4.42	100	strabismic amblyopia
19	0.7	1.52	124	anisometropic amblyopia
19	0.5	1.51	120	anisometropic amblyopia
19	0.8	1.46	107	anisometropic amblyopia
19	1.7	2.33	93	strabismus amblyopia
36	2	5.5	111	anisometropic amblyopia
19	1.3	4.41	120	strabismic/anisometric amblyopia
19	1.4	2.34	112	strabismic amblyopia
18	0.5	10.2	110	anisometropic amblyopia
18	1	4.7	106	anisometropic amblyopia
11	0	15.4	107	normal
9	0	13.8	110	normal
17	0	14.5	105	normal
13	0	14.4	104	normal
12	0	21.9	105	normal
8	0	19.7	102	normal
15	0	13.7	101	normal
36	0	13.2	102	normal
25	0	13.1	105	normal
42	0	10.1	104	normal
12	0	12.2	105	normal
8	0	17.5	107	normal
18	0	11.1	108	normal
12	0	13.6	107	normal
12	0	21	100	normal
9	0	19.4	105	normal
15	0	14.1	103	normal
35	0	19.3	103	normal
27	0	13.9	105	normal
40	0	8.5	103	normal

*BCVA* : best corrected visual acuity.

**Figure 1:**
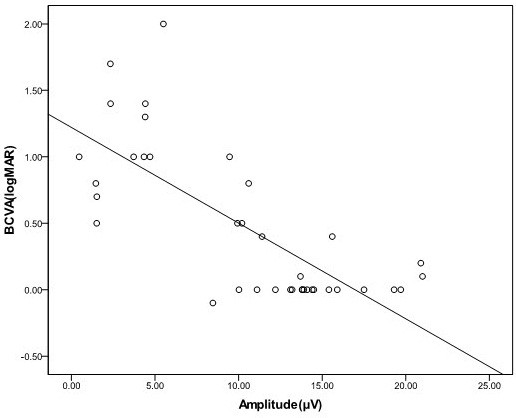
**The correlation between amplitude and visual acuity of normal and amblyopic eyes in pattern VEP.** Pattern amplitude is plotted as a function of visual acuity. The solid line represents the linear regression line fit the data (y = -0.072x + 1.22), and it was statistically significant with p < 0.0001.

There was a significant correlation between amplitude and visual acuity in normal and amblyopic subjects (r = − 0.7649, *p* < 0.0001; Figure 1). Linear regression analysis of pattern amplitude and visual acuity in normal and amblyopic patients indicated an relationship of y = − 0.072*x* + 1.22 (−0.072) (x: amplitude, y: logMAR VA), which was statistically significant.

There was no significant correlation between latency period and visual acuity in normal subjects or amblyopic subjects (r = 0.191, *p* = 0.25) Therefore, linear regression analysis of pattern latency was not performed.

### Predicting visual acuity in patients with optic neuritis

**Figure 2:**
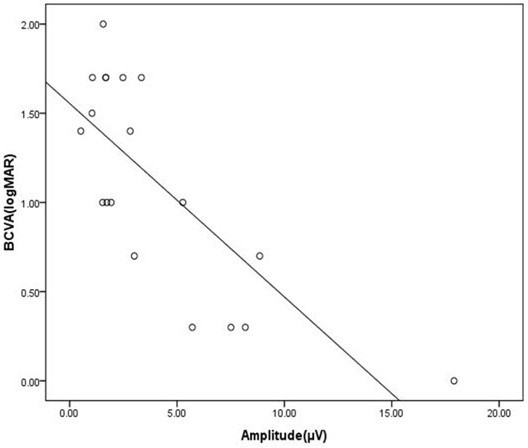
**The correlation between amplitude and visual acuity of optic neuritis in pattern VEP.** Pattern amplitude is plotted as a function of visual acuity. The correlation coefficient was -0.762, p = 0.0002, showing significant relation (*y* = -0.108x + 1.55).

The average amplitude values of 19 optic neuritis eyes was 4.09 ± 4.20 μV, and correlation coefficient between amplitude and visual acuity relationship was −0.762, *p* = 0.0002 (p < 0.01), indicating a significant relationship (Figure 2). Linear regression analysis of pattern amplitude and visual acuity (logMAR acuity) in optic neuritis subjects indicated a relation of y = − 0.108x + 1.55, which was statistically significant.

**Figure 3:**
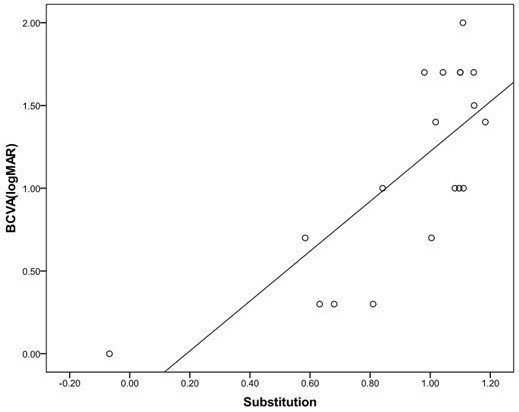
**The correlation between actually measured visual acuity and calculated visual acuity.** The visual acuity calculated by substituting the amplitude value of patients with optic neuritis into the function was 0.93 ± 0.30. The difference in the average between actually measured visual acuity and function value was 0.18 ± 0.4, indicating no statistically significant difference (*t* test, *P* = 0.07).

The average value was calculated by substituting the amplitude value of patients with optic neuritis into the function y = − 0.072x + 1.22 were 0.93 ± 0.30. The difference in the average between actually measured visual acuity and function value was 0.18 ± 0.4, indicating no statistically significant difference (*t*-test, *p* = 0.07; Figure 3).

Another way to determine if the function (y = −0.072x +1.22) is fitted to predict visual acuity in optic neuritis is to perform a comparison of linear regression. We compared function of normal /unilateral amblyopia (y = −0.072x +1.22) vs optic neuritis(y = 0.108x +1.55). The slopes were not significantly different (p > 0.1356). The pooled slope equals −0.0789258 (GraphPad Prism).

### Predicting visual acuity in patients with visual pathway lesions

Linear regression analysis of pattern amplitude and visual acuity (logMAR) in normal and amblyopic patients was useful in predicting visual acuity in optic neuritis. Therefore, we calculated the pattern VEP-estimated VA of 16 eyes of 10 patients to diagnose the presence or absence of visual disability using relationship of y = − 0.072x +1.22 (x: amplitude, y: logMAR acuity) (Table 2). We input the amplitude (N1-P2) of the disability patient in x and obtained y as pattern VEP-estimated VA (logMAR), which was converted to Snellen acuity.

**Table 2:** Clinical characteristics of 16 eyes in 10 patients with visual pathway lesion for disability registry

**Case**	**Eye**	**BCVA**	**Optic disc**	**P100**	**N1-P2**	**-0.072X(N1-P2) + 1.221 Pattern VEP–estimated VA (Log MAR)**	**Pattern VEP–estimated VA (Snellen VA)**
M/43	1	20/2000	Slight pale	113	1.06	1.14	20/276
F/75	2	20/20000	Pale	112	1.27	1.14	20/276
	3	20/2000	pale	110	1.1	1.14	20/276
M/48	4	20/2000	pale	113	2.4	1.05	20/224
	5	20/2000	pale	149	2.52	1.05	20/224
M/36	6	LP(+)	Pale	109	0.65	1.17	20/296
	7	LP(+)	Pale	100	1.78	1.09	20/246
M/48	8	20/2000	Pale	105	5.39	0.83	20/135
	9	20/2000	Pale	122	5.22	0.84	20/138
M/41	10	20/20000	Pale	99	0.27	1.2	20/317
M/66	11	LP(-)	Pale	103	0.76	1.17	20/296
M/53	12	20/200	c/d 0.6	117	4.25	0.91	20/163
	13	20/200	c/d 0.5	112	5.71	0.81	20/129
F/17	14	20/200	Pale	83	1.93	1.08	20/240
M/53	15	20/100	c/d 0.7	114	2.58	1.03	20/214
	16	20/30	c/d 0.8	132	7.61	0.67	20/94

VEP: visual evoked potential, BCVA : best corrected visual acuity, VA: visual acuity, FC : finger counting, HM : hand motion, LP: light perception, C/d : cup/disc.

We attempted to determine a cutoff value for the function that could be applied because the data were limited to very low amplitude. To assess the diagnostic validity of the test, plots of sensitivity vs. specificity, as a Receiver Operator Characteristic (ROC) curve, were made. The most useful cutoff points were found to be at sensitivity (92%), specificity (60%), and accurate screening measure when using cutoff VEP amplitude of 5.77 μV (Table 3). The relationship of y = − 0.072x + 1.22 will be useful to >5.77 μV (<0.8 logMAR).

**Table 3:** Diagnostic validity (sensitivity, specificity) at each cut off point

**Amplitude**	**Sensitivity %**	**Specificity %**
Cutoff (V)		
-8.735	0.0	96
-3.890	0.0	92
-0.420	0.0	80
0.665	7.7	80
1.080	15.4	80
1.185	23.1	80
1.600	30.8	80
2.165	38.5	80
2.460	46.2	80
2.550	53.9	80
2.820	61.5	80
3.655	61.5	60
4.735	69.2	60
5.305	76.9	60
5.550	84.6	60
5.770	92.3	60
6.720	92.3	52
8.805	100	52
10.70	100	36
14.1	100	28

The most useful cutoff points were found to be at sensitivity (92%), specificity (60%), and accurate screening measure when using cutoff VEP amplitude of 5.77 μV.

The severe low amplitude (<5.77 μV) will not provide objective visual acuity measurements. However patients with VEP amplitude < 5.77 μV would be compatible with legal blindness [< 20/200 (1logMAR) VA] required for disability registration.

There was no disagreement for evaluating cases 1 to 11 and 14 as legally blind because of obvious pale optic disc and VEP amplitude ranging from 0.27 μV to 5.39 μV (Table 2).

**Figure 4:**
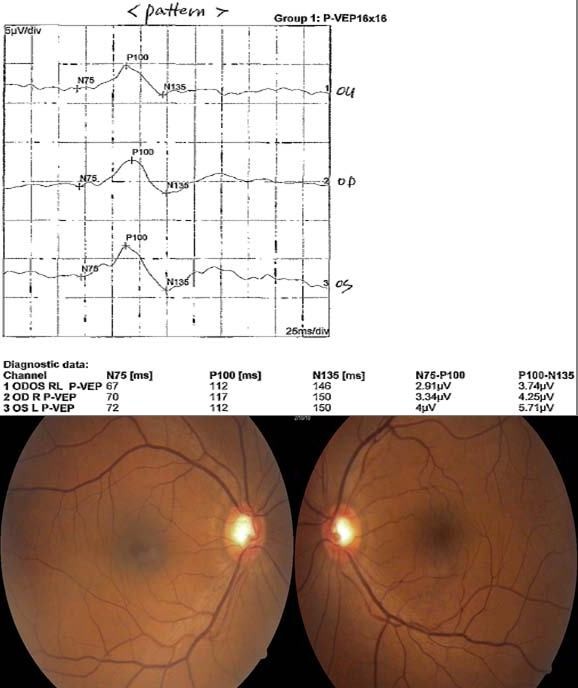
**Pattern VEPs and fundi 6 months following skull fracture in a 53-year-old male.** Note that the similar amplitude and latency in both eye eyes. Visual acuities were 20/200 right; 20/200 left. The optic cup disc ratio was 0.6 rights and 0.5 left.

**Figure 5:**
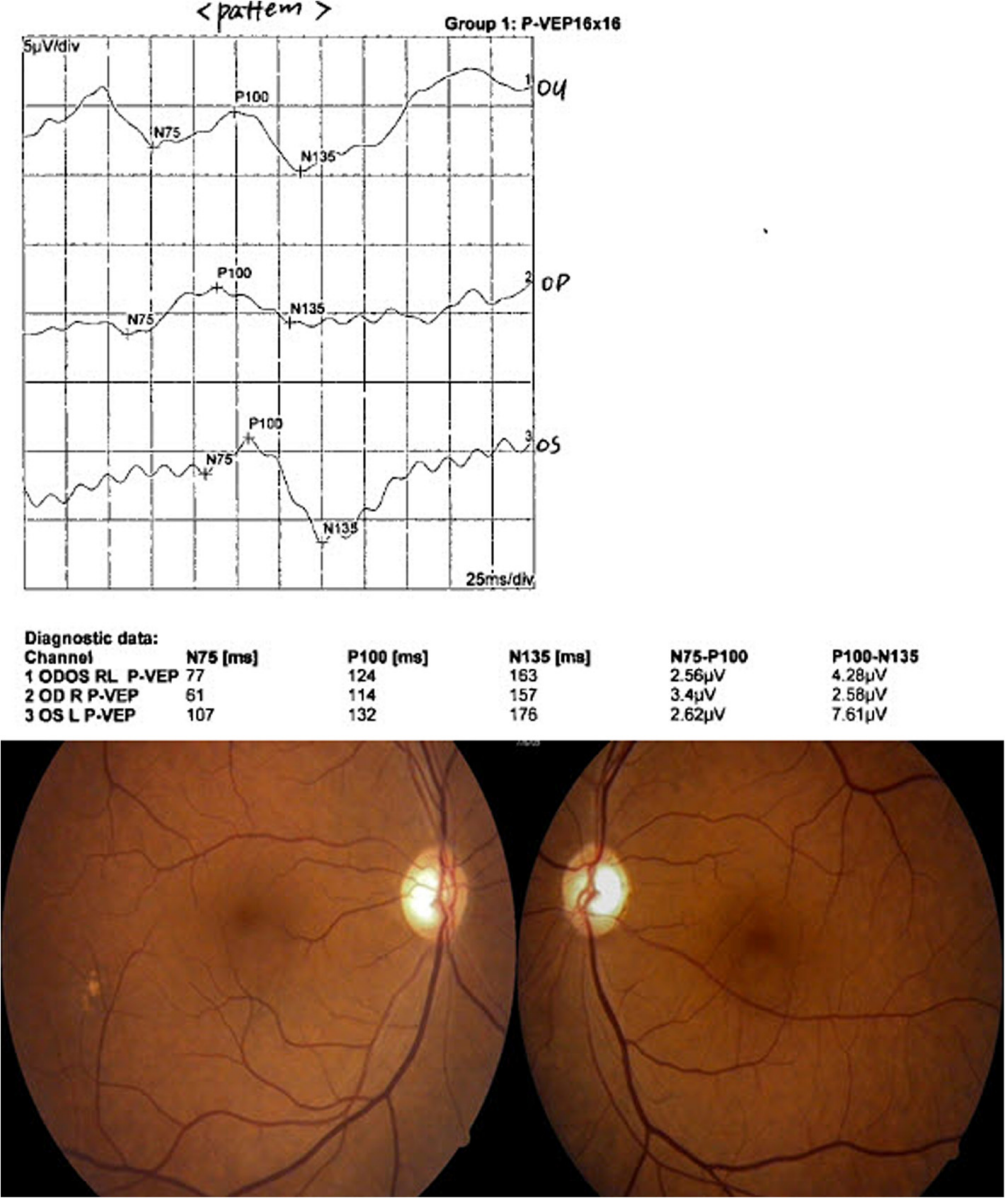
**Pattern VEPs and fundi 1 year following traffic accident in a 53-year-old male.** Note that the similar amplitude in both eye and delayed latency in left eye. Visual acuities were 20/100 right; 20/30 left. Optic disc was temporally slight pale in both eyes.

Clinically, it was challenging to confirm VA in cases 12, 13, 15, and 16, because no definite pale optic discs were noted. This relationship with cutoff amplitude will aid in reliable objective visual acuity assessments, especially in these cases (Figures 4 and 5).

#### Case 12, 13

A 53-year-old male presented complaining of loss of vision in both eyes. He had multiple fractures, including skull fracture from a car accident 6 months prior. He had surgery for fracture of a femur. The patient had no significant prior ocular or systemic history and reported no complaints in vision before the accident. The patient’s visual acuities were O.U. 20/200. The pupil was reactive to light in both eyes. No afferent pupillary defect was noted O.U. Ocular motilities were full. Intraocular pressures were 15 mm Hg O.D. and 16 mm Hg O.S., measured via applanation tonometry. Anterior segment evaluation was normal. Optic cup disc ratio was 0.6 in O.D and 0.5 in O.S. He came to the clinic for evaluation of disability for private insurance. He had poorer vision than we expected. VEP, visual field, and FAG measurements were done. Humphrey 30-2 visual fields were total scotoma in both eyes. FAG was normal. VEP showed similar delayed latency and amplitude in both eyes. The presumed diagnosis of traumatic optic neuropathy was suspected (Figure 4).

The amplitude was below cutoff (4.25 μV, 5.71 μV) in this case. The VEP-estimated VA was estimated as < 20/120 Snellen VA (corresponding to amplitude 5.77 μV). He was diagnosed as legally blind in his both eyes. He was not malingering.

#### Case 15, 16

A 53-year-old male presented complaining of loss of vision in both eyes. He had a traffic accident 1 year prior. He had surgery for a facial bone fracture. The patient’s visual acuities were 20/100 O.D and 20/30 O.S. The pupil was reactive to light in both eyes. No afferent pupillary defect was noted O.U. Ocular motilities showed mild limitation of right and left gaze in both eyes. Intraocular pressures were 10 mm Hg O.D. and 11 mm Hg O.S., measured via applanation tonometry. Anterior segment evaluation was normal. Optic cup disc ratio was 0.7 in O.D and 0.8 in O.S. Optic discs were slightly pale temporally in both eyes. He came to the clinic for evaluation of disability for national disability insurance. He had better vision than we expected. However, his cognitions were unstable. VEP showed mild delayed latency and decreasing amplitude in both eyes (Figure 5).

The reliable pattern VEP -estimated VA of the left eye (7.61 μV) from the correlation was 0.67 logMAR (20/94) O.S because the amplitude was above cutoff (Table 2). Pattern VEP -estimated VA of his right eye (2.58 μV) was <20/120 Snellen VA (corresponding to amplitude 5.77 μV), in spite of unstable cognition. He was diagnosed as legally blind in his right eye. He was not malingering.

## Discussion

This study was performed to examine the usefulness of visual acuity prediction using absolute values, with no comparison between the two eyes of the same subject in one tertiary clinic, in order to diagnose the presence or absence of visual disability. Objective visual acuity is necessary to grade the disability patient. The relationship between amplitude and logMAR acuity is linear even though the VA and amplitude of the data we collected from 2007 to 2009 in amblyopic groups were much lower and not evenly distributed. Thus, a causal distribution of the data was in two clusters (Figure 1). However, the results indicated a statistically significant functional correlation between pattern VEP amplitude and visual acuity in normal vs amblyopic subjects.

We applied the obtained relationship to the optic neuritis patients to see if it is possible in disability patients to differentiate malingering. The pattern VEP- estimated VA was not different from the actually measured subjective visual acuity in optic neuritis cases. The slopes are not significantly different by comparison of linear regression between normal/amblyopia and optic neuritis, so this correlation can be used for evaluating the presence or absence of visual disability.

We had difficulties in evaluating the patients with relatively healthy optic discs in order to measure objective visual acuity for the evaluating the presence or absence of visual disability. An evaluation reference at the clinic is needed. We analyzed this relationship (y = −0.072x + 1.22) to find a reliable cutoff that could be applicable to estimate VA by the ROC curve (area 0.7169, p < 0.03011). Above 5.77 μV amplitude it would be reasonable to predict visual acuity by regression relationship. It is not possible to predict pattern VEP -estimated VA below 5.77 μV (corresponding to 0.8 logMAR: 20/120 Snellen acuity using function y = −0.072x +1.22) because of a very low amplitude.

However, patients with VEP amplitude below 5.77 μV would be compatible with legal blindness [visual acuity below 20/200 (1logMAR)] that is required for disability registration. Visual acuity below 20/200 (1logMAR) is defined as 100% visual acuity loss in visual disability assessment. Suspicious malingering with no obvious pale disc appearance can be ruled out if the amplitude is below 5.77 μV. Our results demonstrated that pattern VEP- estimated VA was useful in confirming subjective visual acuity in disability evaluation at given cutoff amplitude.

Patients with visual disability may exaggerate their decrease in visual acuity of the injured eye to maximize compensation. Clinicians have difficulty confirming visual acuity with visual pathway lesion without apparent pale optic disc. Such patients are particularly difficult to ascertain, and an objective measure of the VA will provide an important contribution to the evaluation of such cases. Electrophysiological testing can be used to evaluate the level of underlying organic dysfunction in patients with nonorganic overlay superimposed upon real dysfunction [13,15-17]. Pattern VEP may be a useful tool for determining the level of visual acuity [18]. Accordingly, cases where visual disability is accompanied by a history of trauma or in patients with amblyopia of definite etiology or bilateral visual loss, the correlation derived from VEP can be used as a reference value for visual acuity. The VEP has been used primarily for objective evaluations of visual acuity and refractive error, but it is impossible to accurately quantify visual function in amblyopia and cases of organic pathology with visual dysfunction.

Odom et al [19] compared subjective and VEP acuity of adults. Linear regression analyses gave good agreement between their subjective data and VEP visual acuity. Our study also showed a significant relationship between VEP amplitude and visual acuity.

This study had some limitations that should be taken into consideration. First, a relatively small number of variables were used, and further studies are needed for verification. However, in our study the range of VEP amplitude was 8-22 μV with 16’ check size in the normal group with 20/20, similar to previous reports [18]. The further studies are needed for verification. Second, VEP amplitude will be affected by other causes of visual acuity loss because the pattern reversal visual evoked response (PVER) mainly represents the function of the macula and optic nerve [20-22]. However, we tried to exclude patients with disability registry with retinal disease by reviewing the results of multifocal ERG and FAG. Third, there has been controversy about the relationship between amplitude of VER and age [23,24]. The unilateral amblyopia group in our study consisted of patients requiring exam for entering the army. Therefore, they were all about 20 years of age. We should consider age differences affecting VEP in future studies. Fourth, the statistical result of optic neuritis in the difference between actually measured visual acuity and function value was marginal (p = 0.07). This non-significant difference was 0.18 logMAR, which is approaching the clinically significant level of 2 lines of letters on a logMAR chart, might be due to a lack of statistical power and/or high levels of variability in their data.

## Conclusion

In conclusion, estimation of visual acuity in visual disability assessment through correlation of absolute amplitude values in pattern VEP might be useful for giving reference visual acuity associated with malingering vs. real disability in some ranges (>5.77 μV). Further studies are required to assess the reliability and to perform statistical verification with additional variables to provide support for use of this method in the assessment of cases of visual disability with visual pathway dysfunction.

## Competing interests

The authors declare that they have no competing interest.

## Authors’ contributions

JHJ performed the statistical analysis and drafted the manuscript. SEK examined and evaluated the patients. SEK and SYO wrote the final manuscript. All authors read and approved the final manuscript.

This work was supported by the research fund of Dankook University in 2010.

## Pre-publication history

The pre-publication history for this paper can be accessed here:

http://www.biomedcentral.com/1471-2415/12/36/prepub
